# Labor Resource Use for Endoscopic Gastric Cancer Screening in Japanese Primary Care Settings: A Work Sampling Study

**DOI:** 10.1371/journal.pone.0088113

**Published:** 2014-02-11

**Authors:** Rei Goto, Kohei Arai, Hirotsugu Kitada, Kazuei Ogoshi, Chisato Hamashima

**Affiliations:** 1 Graduate School of Economics, Kyoto University, Kyoto, Japan; 2 The Hakubi Center of Advanced Research, Kyoto University, Kyoto, Japan; 3 Faculty of Social and Information Studies, Gunma University, Gunma, Japan; 4 Faculty of Business Administration, Hosei University, Tokyo, Japan; 5 Niigata Cancer Center Hospital, Niigata, Japan; 6 Research Center for Cancer Prevention and Screening, National Cancer Center, Tokyo, Japan; Veterans Affairs Medical Center (111D), United States of America

## Abstract

**Objective:**

Endoscopic gastric cancer is screened in primary care settings, but how much resources are required to deliver this service remains unknown. This study determines how much time and human resources are used for endoscopic gastric cancer and for each component of the procedure.

**Materials and Methods:**

Upper endoscopic procedures were prospectively observed using a work sampling technique. This study analyzed data from patients who underwent upper endoscopic gastric cancer screening at primary care clinics that provide this service. The main outcome measurements were time intervals and total time intervals that considered the numbers of simultaneously engaged workers and were calculated as the product of time intervals and the number of workers, and the labor cost of individual components of each procedure.

**Results:**

We observed 44 upper endoscopic procedures at four outpatient clinics. Pre-procedure (preparation and pre-medication), procedure (from intubation to extubation) and post-procedure (recovery and cleaning) accounted for 34.1%, 10.6% and 54.4% of the total time, respectively. Of the overall total time intervals (mean: 4453 person-seconds), 29.3%, 14.4% and 55.7% of the total time was devoted to pre-procedure, procedure and post-procedure, respectively. The post-procedure was the most time- and labor-consuming component from the viewpoints of both total time and labor cost.

**Conclusions:**

Most of the time taken to complete endoscopic gastric cancer screening is consumed by preparation, pre-medication and post-procedures in which nurses play key roles.

## Introduction

Gastric cancer was the second most common cause of cancer death in Japan during 2011 [1]. Since 1983, the Japanese government has sponsored a mass screening program with photofluorography. Guidelines for gastric cancer screening that were developed in 2006 recommend photofluorography for population-based screening [2].

Upper-gastrointestinal endoscopy is another screening method that is routinely applied in Japanese hospitals and out-patient clinics, and several municipalities have provided financial support for endoscopic screening programs. If endoscopic screening can be proven to effectively reduce cancer mortality rates, programs will be introduced for population-based screening. If so, then the ageing population and the diffusion of technological endoscopy are likely to foster an increase in the demand for endoscopic screening. However, an effective cancer screening program is required that is within the constraints of budgets and regional medical labor availability.

How workers use time for each process can be determined and then resources can be efficiently allocated within manufacturing and service industries. Healthcare has also begun to use time studies to evaluate the activities of physicians and nurses [3]. Although endoscopic departments in hospitals have improved internal processes using this method, only a few studies of these issues have been published [4] and details of endoscopic procedures in the primary care setting are unknown. Furthermore, the literature has not described the activity of nurses or how they devote time to procedures.

The present study aimed to clarify workflow processes during upper endoscopic screening programs particularly in the primary care setting to improve the productivity of such programs.

## Methods

### Work sampling method

We assessed the resources used in upper endoscopic gastric cancer screening using work sampling. This statistical method of analyzing work activities allows estimates of the proportions of time dedicated to various elements of work [5]. Since work sampling as a method for measuring work efficiency can evaluate the activities of multiple participants, non-repetitive work cycles and long cycle times, not only manufacturing processes with conveyer lines but more complex activities such as banking operations, research and development management, and healthcare services have been assessed using this method [6]. Endoscopic assessments proceed concurrently with other clinical practices in most Japanese primary care settings. The work cycle of endoscopic procedures is not repetitive and continuous because it is influenced by other activities. In this situation a sampling method is less tiresome and tedious for observers than direct and continuous time-motion studies, although accuracy might be limited within a given degree of statistical validity. We selected the sampled clinics theoretically rather than randomly to understand the structure and efficiency of endoscopic practice.

### Study locations

This study proceeded at four primary care clinics in Niigata City, which is the prefectural Capital of Niigata Prefecture with a population of 811,000. The age-adjusted mortality rate of gastric cancer in Niigata Prefecture is 12.9 per 100,000 persons, which is slightly higher than the national rate of 11.0 per 100,000. Patients in Japan can freely choose between attending a physician's clinic or a hospital. All such clinics profess a specialty and also provide primary care [7]. A clinic offering a gastroenterology specialty is usually equipped with a system for upper endoscopy. Thus, this procedure is routinely applied in primary care settings.

Some municipalities including Niigata city provide financial support for endoscopic cancer screening. Residents of Niigata city who are aged 40, ≥45 or ≥50 years can access endoscopic gastric cancer screening with some out-of-pocket expense according to age and insurance status. Those who are not the targets of this program can also access endoscopic procedures under the social health insurance system, irrespective of symptoms [8].

### Work sampling tools

Activities in a work sampling analysis are recorded in a tabular or matrix form in which each activity falls under several categories. To formulate the work sampling tool, we (RG and KA) preliminarily observed and classified the workflow of two gastrointestinal endoscopic procedures into several components as follows.

- Preparation: Before the first procedure of the day, nurses prepare the endoscope and other needed materials, and conduct operation checks in endoscopy suites and recovery rooms (usually adjoining the suite). Between procedures during the day, nurses change the scope and prepare consumable supplies such as flat sheets, cups and pre-medications.
- Pre-medication: Nurses accompany patients to pre-procedure rooms. Written, informed consent is generally obtained in advance because the patients are usually regular. A nurse explains the procedure and then oral local anesthetic drugs are provided along with other pre-medications such as anticholinergic drugs and deforming agents when deemed necessary. Intravenous (IV) sedation is not used in publicly-supported screening programs. The patients are then transported to the endoscopic suite after a few minutes when the pre-medications have taken effect.
- Procedure: A nurse calls the physician to the room to start the procedure. Biopsies are sometimes performed if pathological findings are suspected.
- Post-procedure: The patients are taken either to a recovery room or to a waiting room, while nurses clean the suite and wash the scope using an automatic washing machine if available. After the last procedure of the day, the endoscope is washed and sterilized with methanol, which takes longer than washing between procedures.

**Figure 1:**
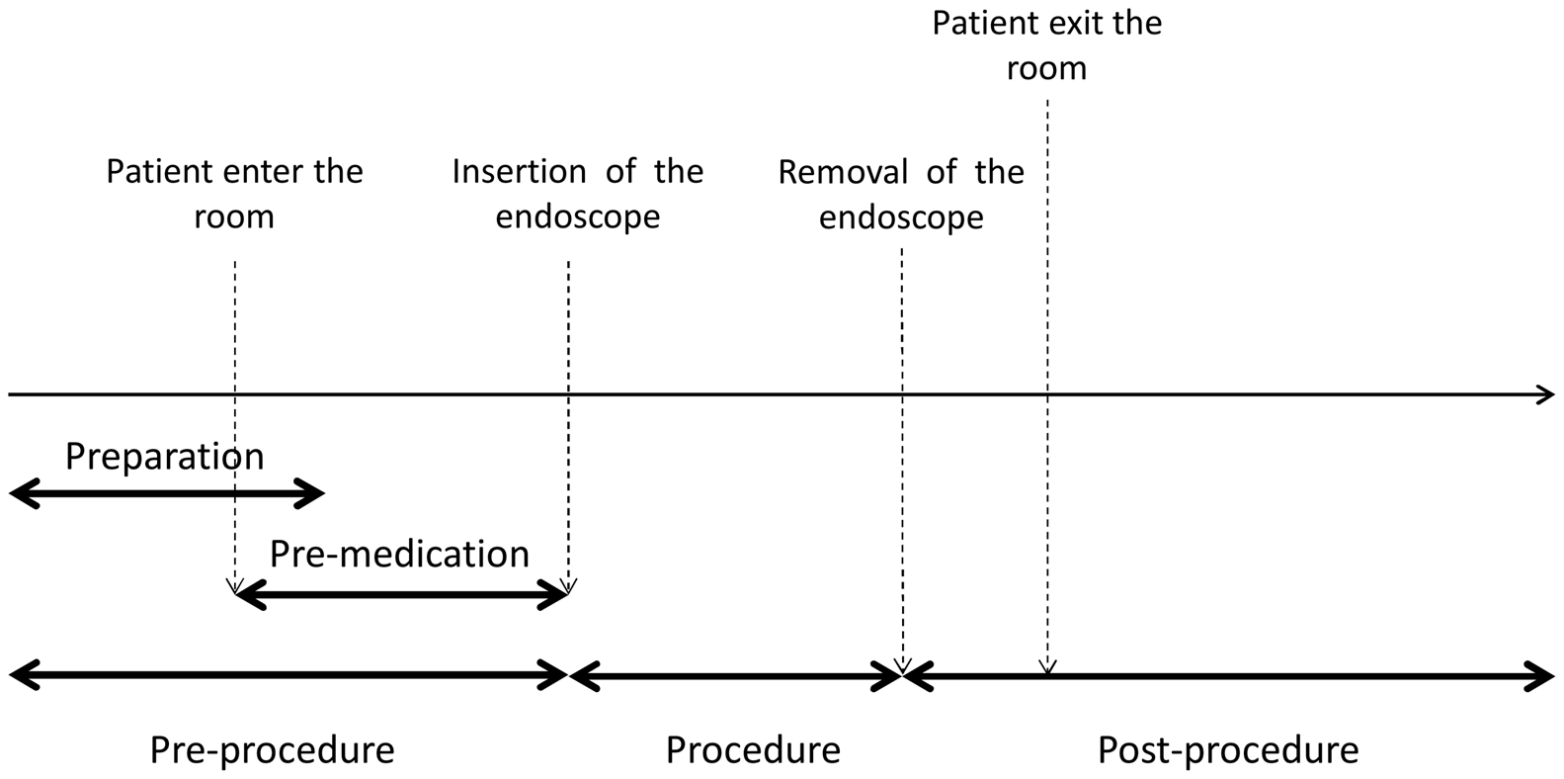
Sequence of endoscopic procedure. Preparation includes setting up endoscope and other materials. During pre-medication, a nurse explains the procedure and local anesthesia, anticholinergic drug and deforming agent are administered. During post-procedure, nurses clean the suite and wash the scope. Physicians participate only in procedure.

Preparation and pre-medication usually overlap or concurrently proceed in separate rooms. We sub-categorized the procedural elements into pre-procedure (preparation+premedication), procedure and post-procedure. Figure 1 shows the time sequence of these categories. The physician participates only in the procedure. Physicians complete a short standardized diagnostic report outside the endoscopy room immediately after the procedure. The results are explained during regular consultations. The time spent by physicians outside the endoscopy room was not included in this analysis.

### Data collection

Data were collected by AK who has a postgraduate degree and HK who is a doctoral candidate specializing in management and work sampling methods. We selected four of the primary care clinics that participated in screening programs supported by the local government. All are solitary practices, which comprise the most popular type of practice in Japan. All procedures were directly observed by a single person who completed a work sampling sheet created based on the procedural components described above. The time when each component started and ended and those who conducted it were recorded. The number and duration of waiting times and discontinuities were also recorded.

The total procedural duration was classified as follows. Processing time constitutes the bulk of the activities of doctors and nurses including setup time and run time. Wait time is defined as the amount of time spent by doctors and/or nurses waiting for others to complete their activities, for example, the time frame necessary for the onset of action and/or running an automatic washer. Idle time refers to activities during this interval other than endoscope-related procedures such as the time taken to examine other patients or the amount of time spent waiting for the patients' bathroom to become vacant. Because endoscopic inspections proceeded in parallel with regular consultations at clinics, we excluded idle time from the analysis and defined total duration as the sum of processing and wait times.

We also recorded the number of staff involved in each procedure. The unit labor cost of physicians and nurses can be calculated using the salaries and working hours reported by the Basic Survey of Wage Structure [9]. The hourly wage of physicians and of nurses is 5,474 JPY (60.8 USD) and 2,340 JPY (26.0 USD), respectively. Total labor time and cost incurred for each component of endoscopic procedures are also reported.

### Data analysis

Differences in time intervals were analyzed using ordinal least squared regression. Age, the use of transnasal endoscopy, simultaneous biopsies and the first and last procedures of the day were assumed to impact time intervals according to the observations. Moreover, random coefficient regression was estimated to allow clinic heterogeneity to affect parameters. We considered a *p* value of <0.05 to be statistically significant.

### Ethics statement

An endoscopist explained the procedure to patients, and then written, informed consent was obtained from all patients. All clinics that participated in this study used the same consent form. The institutional review board at the National Cancer Center approved the present study on October 1, 2010.

## Results

We observed 44 upper endoscopic procedures at four outpatient clinics between August 30 and September 9, 2011. A physician ran the practice and two to four nurses worked at each clinic. The median weekly numbers of outpatient consultations and upper endoscopies were 676 and 19.7, respectively. Table 1 provides descriptive data about of the patients. The mean (SD) age was 63.9±9.2 years, and 16 (36.4%) were male. Our samples included younger and more male patients to be comparable with the age and sex distribution of the entire screened population [10].

**Table 1:** Descriptive statistics.

	Total (n = 44)
Standard deviation of age	9.21
Male patients (%)	36.36
Transnasal Endoscopy (n)	9
Biopsies (n)	2
Mean number of nurses (n)	
re-procedure	1.35
rocedure	1.02
ost-procedure	1.54

Among the four physicians in the present study who performed endoscopic procedures, three were certified as endoscopic specialists by the Japan Gastroenterological Endoscopy Society. Transnasal endoscopy was applied in 9 (20.5%) procedures. At least two nurses usually participated in pre- and post-procedures and one assisted the physician during procedures. Thus, the actual number of involved nurses must be included in estimates of total labor cost, particularly the pre- and post-procedural components.

Table 2 shows time intervals as well as the total time intervals that include the number of simultaneously engaged workers. Total time intervals were thus calculated as the product of time intervals and the number of workers. The mean pre-procedural, procedural and post-procedural time intervals were 1022 316.3 and 1629 seconds, respectively, which accounted for 34.1%, 10.6% and 54.4%, respectively, of the mean total time interval of 2996 seconds. When the first and the last procedures were not required, less time was spent on all three components. The relative standard deviations (SD/mean) were 69.8%, 26.4% and 50.7%, respectively. The time interval of the procedure varied the least among the three components.

**Table 2:** Time intervals of components.

	Time Intervals (s)		Total time intervals (person-seconds)	
	All samples (*SD*, range) n = 44	Samples without First and last of the day (*SD*, range) n = 26	All samples (*SD*, range) n = 44	Samples without First and last of the day (*SD*, range) n = 26
Pre-procedure	1022	923.2	1305	1144
	(713, 140–2922)	(697, 140–2922)	(867, 162–3002)	(778, 162–2922)
Procedure	316.3	304.4	642.8	608.9
	(83.5, 190–535)	(80.3, 190–535)	(193, 380–1311)	(161, 380–1070)
Post-procedure	1629	1449	2482	2066
	(826, 286–3526)	(673, 287–2859)	(1728, 297–7052)	(1351, 317–5718)
Total	2996	2676	4453	3818
	(1262, 735–6460)	(1179, 735–4695)	(2261,1211–10076)	(1929, 1211–7489)

Note: Total time interval is the product of time intervals and number of workers. These intervals reflect number of simultaneously engaged workers and time intervals of each worker.

The mean pre-procedural, procedural and post-procedural total time intervals t were 1305, 642.8 and 2482 person-seconds, respectively, which accounted for 29.3%, 14.4% and 55.7%, respectively, of the mean total time interval of 4453 person-seconds.

Table 3 describes the labor cost of each component. The mean pre-procedural, procedural and post-procedural labor costs were 792 JPY (8.80 USD if 1 USD = 90 JPY), 679.3 JPY (7.55 USD) and 1508 JPY (16.8 USD), respectively, which accounted for 26.5%, 22.7% and 50.4%, respectively' of the mean overall total labor cost of 2991 JPY (33.3 USD). Thus, post-procedure was the most time and labor consuming component, followed by the pre-procedure and procedure from the viewpoints of time, total time or labor cost.

**Table 3:** Labor cost of components.

	Labor cost (JPY; SD, range)
Pre-procedure	792.6 (537, 98.42–1824)
Procedure	679.3 (191, 404.3–1195)
Post-procedure	1508 (1050, 180.4–4284)
Total	2991 (1424, 972.2–6519)

Note: JPY, Japanese Yen.

Table 4 shows the results of the OLS regression of time intervals. The significant intercept shows that some time intervals are needed in each procedure irrespective of confounding factors analyzed here. Transnasal endoscopic screening pre-procedures and procedures take significantly longer, although time intervals over the total procedure did not significantly differ. The age of the patients did not significantly affect time intervals. The first procedure of the day took significantly longer only during the pre-procedure. The final post-procedure and total procedure of the day were both significantly longer. The OLS regression produced rather high adjusted R^2^ values, indicating that these variables can explain fine variations in time intervals. Table 5 shows random coefficient regression at the clinic level. We allowed for stochastic heterogeneity in model parameters for the patients at each clinic, except for age. We adopted this method to determine whether heterogeneity among clinics such as the number of endoscopes, nurses or rooms associated with each procedure would affect the findings shown in Table 4. Although the t values decreased, the results were similar between Tables 4 and 5.

**Table 4:** OLS regression of time intervals.

	Pre-procedure		Procedure		Post-procedure		Total procedure	
	Estimate	t-statistics	Estimate	t-statistics	Estimate	t-statistics	Estimate	t-statistics
Variables								
Intercept	1621.28*	−2.47	187.78*	2.68	1768.49*	2.09	3278.44†	2.95
Age	−7.36	−0.80	2.18*	2.27	2.11	0.18	1.61	0.11
Transnasal dummy	779.88†	3.15	97.49‡	3.71	−71.83	−0.23	857.65*	2.16
Biopsies dummy			143.40‡	3.82			1435.60*	2.36
Dummy variable: first procedure of the day	389.90*	2.07					163.18	0.54
Dummy variable: last procedure of the day					1261.68‡	5.07	842.11*	2.43
Adjusted R^2^	0.5874		0.6451		0.4962		0.6646	
*F*-statistic	10.73‡		13.72‡		7.90‡		11.16‡	

Note: Regression model included dummy variables of clinics (not reported).

* p<.05,

† p<.01,

‡ p<.001.

**Table 5:** Multilevel regression of time intervals.

	Pre-procedure		Procedure		Post-procedure		Total procedure	
	Estimate	t-statistics	Estimate	t-statistics	Estimate	t-statistics	Estimate	t-statistics
Variables								
Intercept	940.85	1.66	288.97‡	3.13	2208.34‡	3.45	2046.01‡	3.18
Age	−2.83	−0.31	−0.03	−0.02	−13.55	−1.43	6.77	0.66
Transnasal dummy	729.26†	2.10	122.56§	8.01	76.38	0.35	1027.96§	5.01
Biopsies dummy			168.44†	2.25			779.43	1.03
Dummy variable: first procedure of the day	244.7	1.38					206.9	0.90
Dummy variable: last procedure of the day					1159.30‡	3.05	779.43*	1.80
AIC	632.79		463.03		662.14			

Note:

* p<.1,

† p<.05,

‡ p<.01,

§ p<.001.

## Discussion

This sampling study investigated the time and labor resources devoted to endoscopic gastric cancer screening in four primary care settings. This method is very helpful for evaluating the activities of physicians and nurses. It has been applied to enhance the efficiency of labor force input from a management perspective and to estimate actual time costs of fundamental information with which to economically evaluate health care [11].

Our main findings are as follows. Firstly, physicians are engaged only in scope intubation and nurses performed all other procedures such as pre-medication preparation and cleaning up. Secondly, procedures during which an endoscope is intubated accounted for about 10% of the time interval of the entire procedure. Thirdly, the post-procedure was the most time and labor consuming component, followed by the pre-procedure and procedure from the viewpoints of total time and labor cost. Fourthly, the pre-procedure and procedure takes significantly longer for transnasal endoscopic screening.

Primary care physicians in Japan are reimbursed under a uniform fee-for-service schedule of public health insurance. Local government contracts with a local medical association using the reimbursement rates for upper endoscopic tests as the benchmark for setting prices. The reimbursement rate for upper-gastrointestinal endoscopy in the 2010 tariff was 11,400 JPY (127 USD). Therefore, the total labor cost of 2,991 JPY (33.3 USD) accounted for 26.2% of the marginal revenue for this procedure. If a biopsy is performed, 3,000 JPY (33.3 USD) is also reimbursed. The total labor cost is increased by adding the amount of time required to complete the biopsy (143.4 seconds). Thus, the total labor cost of 3,301 JPY (36.7 USD) accounted for 22.9% of the marginal revenue for this procedure. The Japan Medical Association Research Institute reported in 2010 that the labor cost is 52.3% of the total revenue in primary care clinics [12]. Endoscopic gastric cancer screening is a less labor-intensive procedure on average.

Nursing is also important in the provision of endoscopic tests. The pre- and post- procedures account for most of the time spent on the entire procedure. The provision of endoscopic services would be more efficient if more than one nurse is involved. However, the shortage of nurses is a social problem in Japan [13]. The procedure, in which physicians are always involved, is the least varied component in terms of time intervals. The actual testing procedure is in fact more standardized than the pre- and post- procedures. This indicates that nurse education and the standardization of nursing activities could offer some room for efficiency improvement. Investment in capital resources to relieve workload could also improve efficiency. Examples are the introduction of automated washing devices for endoscopes during the post-procedure or employing staff to perform such tasks that do not require medical training.

Labor cost is mainly considered in this paper. Other types of cost include consumables such as medicines, syringes and flat sheets, fuel and light, capital items such as endoscopic equipment, and overhead costs such as administration and rent. In general, average capital costs are decreased by the efficient use of capital resources. Harewood et al. [4] suggested several measures to improve turnover in endoscopic suites based on a time-and-motion study. Yong et al. [14] noticed delays in the procedure as an important source of inefficiency. Here, we assessed workflow in primary care settings where other routine medical practices proceed in parallel. To improve the use of capital resources by increasing the number of endoscopic tests might not be feasible if the total number of outpatients is sufficiently high.

This study has several limitations. The representativeness of the data might not be assured in this study, only a few municipalities support endoscopic cancer screening and we collected data from only four clinics in a specific region. We observed time cost only in endoscopy rooms. Physicians provide detailed explanations about screening procedures and obtain written, informed consent and they can prepare documents in advance because the patients are usually regular. They also complete simple diagnostic reports at their workplace. Another time cost concerns administration of the entire screening program. All diagnoses made by endoscopists are confirmed by other specialists after collecting films and image files. Time costs outside the endoscopy room should be considered when discussing the efficiency of the program overall.

We might not have considered some confounding factors. Our observations were derived from screened individuals who were generally healthy. However, some comorbidities might have impacted the results. For example, difficulties in locomotion might prolong pre- and post-procedures regardless of nursing assistance. Variables associated with the experience of physicians and nurses were not fully considered. Further investigations of larger samples are required.

We did not consider clinical outcomes such as the prevalence of complications and other measures of the quality of care. Short time intervals during endoscopic screening tests do not always guarantee good practice.

We did not consider cancer screening in the hospital setting. If hospitals could deliver this procedure more efficiently, providing this service in the hospital setting would be more effective from the viewpoint of healthcare costs. However, primary care clinics offer the advantage of reducing travel and time costs for patients. The institutions that could deliver endoscopic cancer screening programs would depend on the distribution of functions between hospitals and clinics. If regional hospitals must concentrate on high-level care, labor costs could be saved by leaving screening services to primary care clinics.

In conclusion, the present findings highlight the importance of the amount of time consumed during the preparation, pre-medication and post-procedural components of endoscopic gastric cancer screening in which the contribution of nurses is quite important.
